# Copy number polymorphisms and anticancer pharmacogenomics

**DOI:** 10.1186/gb-2011-12-5-r46

**Published:** 2011-05-25

**Authors:** Eric R Gamazon, R Stephanie Huang, M Eileen Dolan, Nancy J Cox

**Affiliations:** 1 Section of Genetic Medicine, Department of Medicine, University of Chicago, 900 East 57th Street, Chicago, IL 60637, USA; 2 Section of Hematology/Oncology, Department of Medicine, University of Chicago, 900 East 57th Street, Chicago, IL 60637, USA; 3 Department of Human Genetics, University of Chicago, 920 East 58th Street, CLSC 5th floor, Chicago, IL 60637, USA

## Abstract

**Background:**

Recent studies have investigated the contribution of copy number variants (CNVs) to disease susceptibility in a multitude of complex disorders, including systemic lupus erythematosus, Crohn's disease, and various neurodevelopmental disorders. Relatively few CNV studies, however, have been conducted on pharmacologic phenotypes even though these structural variants are likely to play an important role. We developed a genome-wide method to identify CNVs that contribute to heterogeneity in drug response, focusing on drugs that are widely used in anticancer treatment regimens.

**Results:**

We conducted a comprehensive genome-wide study of CNVs from population-scale array-based and sequencing-based surveys by analyzing their effect on cellular sensitivity to platinating agents and topoisomerase II inhibitors. We identified extensive CNV regions associated with cellular sensitivity to functionally diverse chemotherapeutics, supporting the hypothesis that variation in copy number contributes to variation in drug response. Interestingly, although single nucleotide polymorphisms (SNPs) tag some of the CNVs associated with drug sensitivity, several of the most significant CNV-drug associations are independent of SNPs; consequently, they represent genetic variations that have not been previously interrogated by SNP studies of pharmacologic phenotypes.

**Conclusions:**

Our findings demonstrate that pharmacogenomic studies may greatly benefit from the study of CNVs as expression quantitative trait loci, thus contributing broadly to our understanding of the complex traits genetics of CNVs. We also extend our PACdb resource, a database that makes available to the scientific community relationships between genetic variation, gene expression, and sensitivity to various drugs in cell-based models.

## Background

Copy number variants (CNVs) have received considerable attention in recent years as studies have implicated them in a wide range of complex human phenotypes, including susceptibility to HIV-1/AIDS [1], Crohn's disease [2], and various autoimmune disorders. The systematic assessment of their role in the etiology of complex disease has been predicated on improvements in genotyping technologies (including SNP-based genotyping arrays and clone-based comparative genomic hybridization) and on advances in algorithms for copy number analysis [3]. Genome-wide surveys of CNVs [4,5] have sought to produce a comprehensive map to enable disease association studies, but a recent comprehensive study reports a somewhat disappointing finding that CNVs are likely to make a relatively minor contribution to the genetic basis of complex traits [6], particularly disease susceptibility.

While the study of the contribution of CNVs to drug response has lagged behind the investigation of their contribution to disease risk, there have been some notable findings coming out of candidate gene approaches. The gene *CYP2D6 *encodes an enzyme to which the metabolism of a large number of drugs, such as antidepressants, neuroleptics, analgetics and anticancer drugs, is attributed. It has been demonstrated that *CYP2D6 *may occur in CNVs of 0 to 13 copies [7]. Studies have shown that copy number for this gene affects the plasma levels of the active metabolite of tamoxifen, namely endoxifen, so that ultra-rapid metabolizers who carry more than two copies of the gene show much higher levels of endoxifen than those who carry the regular copy number for the gene [8]. Higher *CYP2D6 *activity due to gene amplification has also been shown to predispose to life-threatening opioid intoxication [9]. Another drug metabolizing cytochrome P450 gene, *CYP2A6*, also occurs in variable copy number. *CYP2A6 *encodes an enzyme that metabolizes several drugs, including nicotine and its metabolite cotinine. Increased *CYP2A6 *activity has been shown to be responsible for increased risk for nicotine addiction [10] and for tobacco-related cancers. The SULT family of Phase II conjugating enzymes, particularly that encoded by *SULT1A1*, has been the subject of extensive pharmacogenetic studies that show the importance of CNVs as a genetic source of variability in the metabolic activity of these enzymes. SULT pharmacogenomic studies [11] have highlighted CNV-based mechanisms that lead to increased risk for chemical carcinogenesis and adverse drug reactions. Glutathione S-transferase (GST), also a phase II family of conjugation enzymes, plays an important role in the detoxification of drugs. Studies have shown that homozygous deletion of *GSTM1 *is correlated with increased cancer risk and with better treatment outcome [12,13]. These findings and related developments highlight the necessity of incorporating copy number analysis in elucidating the genetic underpinnings of drug response.

The recently released catalog [4] from an extensive survey of copy number regions assayed in cell lines from the International HapMap project and the subsequent study of genomic structural variants based on whole genome DNA sequencing data (the 1000 Genomes Project) [14] allow for new pharmacogenomic discoveries and for deep insights into the genetic basis of pharmacologic phenotypes, which to date has largely been based on studies of SNPs [15]. In whole-genome studies using lymphoblastoid cell lines (LCLs), cellular sensitivity to drug [16] as well as gene expression phenotypes [17] have been shown to be heritable [18] and to include a significant genetic component. Although many CNV pharmacogenetic studies have focused on pharmacokinetic genes, we chose to evaluate pharmacodynamic genes using an LCL-based model. Studies in our laboratory have generated a rich resource of pharmacologic data [19] on a wide array of chemotherapeutic agents using the HapMap cell lines, enabling us to conduct a systematic analysis of the role of CNVs for a variety of anticancer drugs.

## Results

### Genome-wide association studies

LCLs from unrelated CEU samples were phenotyped for cellular sensitivity to the four chemotherapeutic drugs included in our study: carboplatin [20], cisplatin [21], daunorubicin [22], and etoposide [16]. We conducted genome-wide association scans using drug IC_50 _as a quantitative trait.

A total of 5,238 CNVs from an array-based study [4] were evaluated in genome-wide association studies (GWAS) against cellular sensitivity drug phenotypes. Of these CNVs, 77% are deletions (0, 1, or 2 copy number), 16% are amplifications (2, 3, or 4 copy number), and the remainder are multi-allelic (greater than 3 diploid copy number genotypes) [4]. At the nominally significant threshold of *P *< 0.05, we identified 67 CNVs associated with carboplatin IC_50_, 70 CNVs with cisplatin IC_50_, 73 CNVs with daunorubicin IC_50_, and 113 CNVs with etoposide IC_50_.

### Genomic characterization of drug susceptibility-associated CNVs

We further evaluated the genomic characteristics of these drug susceptibility-associated CNVs for their size and type (deletion versus amplification). In general, there is little (Pearson) correlation between the size of a CNV and its association with cellular sensitivity to carboplatin (r = 0.020), cisplatin (r = 0.008), daunorubicin (r = 0.054) and etoposide (r = 0.024). We did, however, observe that the top CNVs associated (*P *< 0.05) with IC_50 _for daunorubicin are significantly smaller (average size of 10.6 kb) than expected (average size of 14 kb) from the full set of CNVs included in our study; etoposide-associated CNVs are, in contrast, close to expectation (average size of 13.4 kb). The CNVs associated with carboplatin and cisplatin IC_50 _(average size of 11.2 kb and 11.4 kb, respectively) are significantly smaller than expected.

Sixty-two of the 67 carboplatin-associated CNVs (*P *< 0.05) are biallelic (that is, deletions or amplifications); the remaining five CNVs are multi-allelic CNVs (that is, defined as having more than three CNV genotypes). Deletions are significantly more frequent (85%) than duplications among the carboplatin-associated CNVs. Similarly, all but 4 of the 70 cisplatin-associated CNVs (*P *< 0.05) are biallelic. The top cisplatin-associated CNVs are significantly more likely to be deletions (87%) than duplications.

All but 9 etoposide-associated CNVs (*P *< 0.05) are biallelic; 69 out of the 73 daunorubicin-associated CNVs (*P *< 0.05) are biallelic. Nearly 82% of the daunorubicin-associated CNVs and 82% of the etoposide-associated CNVs are deletions.

### Drug susceptibility-associated CNVs are enriched for expression quantitative trait loci

We observed that no exons overlap the genomic regions defined by the top associated CNVs for each anticancer drug included in our study, suggesting that these CNVs do not act to disrupt coding sequence. We thus hypothesized that these CNVs act to influence gene regulation. We evaluated the functional import of the drug susceptibility-associated CNVs by considering their effect on global gene expression. At an expression association threshold of *P *< 0.0001, 60% (*N *= 40) of the top CNVs associated with carboplatin (*P *< 0.05) were found to be expression quantitative trait loci (eQTLs). Interestingly, two of the top carboplatin-associated CNVs (*CNVR3882.1 *on chromosome 8 and *CNVR666.1 *on chromosome 2) predict the expression of *SELL*. We found that *SELL *expression level is also significantly correlated with carboplatin IC_50 _(*P *= 0.02) in the CEU samples. We identified several target genes of carboplatin-associated CNVs (as eQTLs) whose expression levels were significantly correlated (after multiple testing correction [23], false discovery rate (FDR) <0.05) with carboplatin IC_50, _including *PHGDH*, *MYO1B*, *TGFBR2*, and *PRF1*. Similarly, nearly 56% (*N *= 39) of the cisplatin-associated CNVs (*P *< 0.05) were associated with the transcript level of genes as eQTLs. We found a target gene, *MAST4*, for two cisplatin-associated CNVs (*CNVR2968.1 *on chromosome 6 and *CNVR7881.1 *on chromosome 20). *MAST4 *trends toward significance (*P *= 0.06) with cisplatin IC_50 _in the CEU samples. A target gene (*C4A *at *P *= 8.2 × 10^-6^) for a cisplatin-associated CNV eQTL (*CNVR4748.1 *on chromosome 10) was found to be significantly correlated (after multiple testing correction [23], FDR <0.05) with cisplatin IC_50_.

Restricting our analysis to biallelic CNVs, we found, through simulations, that the top CNVs, for each platinating agent, are significantly enriched for eQTLs relative to frequency-matched SNPs (enrichment *P *< 0.05). The eQTL enrichment holds at a lower *P*-value threshold (*P *= 10^-6^) used to define an eQTL, showing the robustness of our observation to the definition of eQTL. See Materials and methods for details on the simulation procedure.

Of the top CNVs associated with etoposide IC_50 _(*P *< 0.05), 76% (*N *= 86) were found to be eQTLs. Of these CNV eQTLs, eight share *UBA1 *as a target gene (*P *< 9.7 × 10^-5^). Two target genes (*PLEKHG6 *at *P *= 1.3 × 10^-6 ^and *WSB2 *at *P *= 8.0 × 10^-5^) for etoposide-associated CNV eQTLs (*CNVR4784.1 *on chromosome 10 and *CNVR1874.1 *on chromosome 4, respectively) were found to be significantly correlated with etoposide IC_50 _(FDR < 0.01) [23]. Nearly 52% (*N *= 38 of the top CNVs associated with daunorubicin IC_50 _(*P *< 0.05) were eQTLs. We identified two daunorubicin-associated CNVs (*CNVR479.2 *and *CNVR332.1 *on chromosome 1) predicting the expression of *HIST1H4A *(*P *< 6.7 × 10^-5^); we also found the expression level of this gene to be correlated (*P *= 3.7 × 10^-8^) with daunorubicin IC_50 _in the CEU samples. We identified several target genes (including *PAPLN *at *P *= 3.3 × 10^-5 ^and *KLF12 *at *P *= 6.1 × 10^-5^) for daunorubicin-associated CNV eQTLs (*CNVR2616.1 *on chromosome 5 and *CNVR948.1 *on chromosome 2, respectively) whose expression levels were significantly correlated (after multiple testing correction [23], FDR < 0.05) with daunorubicin IC_50_.

As in the case of the platinating agents, we found, through simulations, that the top CNVs for each topoisomerase II inhibitor are more likely to be eQTLs than frequency-matched SNPs (enrichment *P *< 0.05).

### Functional characterization of transcripts *cis*-regulated by deletions from whole genome sequencing data

**Figure 1:**
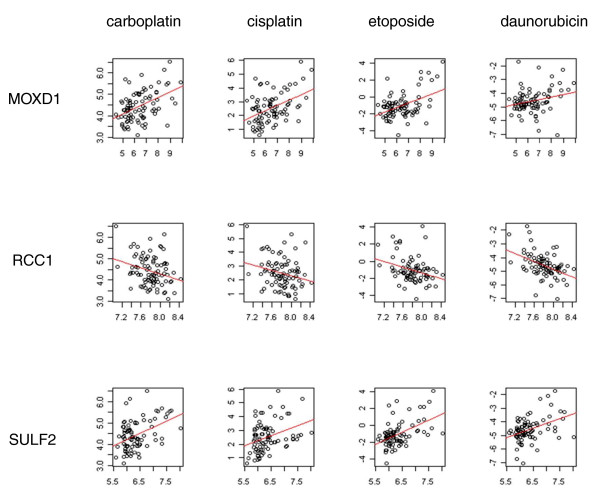
**Three transcripts *cis*-regulated by CNVs identified by whole genome sequencing data predict cellular sensitivity to functionally diverse chemotherapeutics**. Through ENCODE/Gencode annotation, 376 genes were recently identified [12] to be *cis*-regulated by CNV deletions. At *P *< 0.05, we found 21 gene expression correlations with carboplatin, 15 with cisplatin, 23 with daunorubicin, and 21 with etoposide. Three transcripts (*MOXD1 *on chromosome 6, *RCC1 *on chromosome 1, *SULF2I *on chromosome 20) were significant after multiple testing adjustment (p_*adj *_< 0.05) and were the only CNV deletions associated with all four agents at the nominal *P *< 0.05 threshold. The plots show level of expression (x axis) and IC_50 _(y axis).

Given the observed high proportion of deletions among CNVs associated with cellular sensitivity to chemotherapeutic agents, we sought additional functional support for the role of CNVs as transcriptional regulators from whole genome sequencing data coming out of the 1000 Genomes project, which characterized the CNV deletions with Gencode/ENCODE transcripts [14]. The resulting enlarged catalog of CNVs (with an initial focus on deletions) included CNVs of size 50 bp or larger mapped at single nucleotide resolution. We identified 376 transcripts to which CNV deletions were annotated [14] (by Gencode/ENCODE) as influencing (*cis*-regulating) transcription and/or translation. We proceeded to test the 376 transcripts for their role in predicting cellular sensitivity to chemotherapeutics. At *P *< 0.05, we found 21 transcript correlations with carboplatin, 15 with cisplatin, 23 with daunorubicin, and 21 with etoposide (see Table 1). Three transcripts (*MOXD1*, *RCC1*, *SULF2*) were significant after multiple testing adjustment (p_*adj *_< 0.05, Bonferroni). Remarkably, the three transcripts were the only CNV deletions associated with all four agents at the nominal *P *< 0.05 threshold (Figure 1).

**Table 1:** Nominally significant (*P *< 0.05) gene expression correlations with cellular sensitivity to chemotherapeutic agents

Gene	Drug	*P*-value	Chromosome
*AKT1S1*	Carboplatin	0.04497	19
*AKT1S1*	Daunorubicin	0.01149	19
*AKT1S1*	Etoposide	0.00610	19
*ALKBH8*	Daunorubicin	0.01151	11
*AMY1A*	Etoposide	0.03933	1
*AMY2A*	Etoposide	0.03933	1
*ANKLE1*	Carboplatin	0.03017	19
*ANKLE1*	Daunorubicin	0.01706	19
*ANKRD36B*	Daunorubicin	0.02380	2
*BCLAF1*	Daunorubicin	0.01498	6
*C13orf3*	Carboplatin	0.00830	13
*C13orf3*	Cisplatin	0.01980	13
*C13orf3*	Daunorubicin	0.00572	13
*C18orf1*	Cisplatin	0.00398	18
*CAB39L*	Etoposide	0.04179	13
*DIS3L2*	Daunorubicin	0.03077	2
*DNAJC5*	Carboplatin	0.00166	20
*DNAJC5*	Cisplatin	0.04058	20
*FBRS*	Carboplatin	0.00661	16
*FGFR4*	Carboplatin	0.00556	5
*FKBP14*	Carboplatin	0.03912	7
*FLI1*	Carboplatin	0.04735	11
*FLI1*	Daunorubicin	0.00314	11
*FLI1*	Etoposide	0.04230	11
*GALNT1*	Daunorubicin	0.00065	18
*GPR107*	Etoposide	0.00674	9
*GPR137*	Carboplatin	0.00616	11
*GPR137*	Cisplatin	0.00828	11
*GPR137*	Etoposide	0.01493	11
*GPR144*	Etoposide	0.04526	9
*GSPT1*	Carboplatin	0.04002	16
*GSPT1*	Cisplatin	0.01429	16
*HLA-DQA1*	Daunorubicin	0.00946	6
*HLA-DQA1*	Etoposide	0.00022	6
*IGLV3-21*	Carboplatin	0.04800	22
*MOXD1*	Carboplatin	0.00002	6
*MOXD1*	Cisplatin	0.00002	6
*MOXD1*	Daunorubicin	0.03454	6
*MOXD1*	Etoposide	0.00007	6
*MTA1*	Etoposide	0.01401	14
*NCOA1*	Daunorubicin	0.04278	2
*NEK6*	Carboplatin	0.04050	9
*NUB1*	Etoposide	0.02671	7
*PPP1R3B*	Cisplatin	0.03039	8
*PTP4A2*	Carboplatin	0.01930	1
*PTP4A2*	Cisplatin	0.01182	1
*PTP4A2*	Etoposide	0.03348	1
*RCC1*	Carboplatin	0.01252	1
*RCC1*	Cisplatin	0.01722	1
*RCC1*	Daunorubicin	0.00002	1
*RCC1*	Etoposide	0.01358	1
*RNF103*	Daunorubicin	0.02655	2
*RPN2*	Daunorubicin	0.01206	20
*SLC10A7*	Daunorubicin	0.00481	4
*SLC4A8*	Cisplatin	0.02189	12
*SLC4A8*	Etoposide	0.00248	12
*SMPD4*	Daunorubicin	0.02446	2
*SNRK*	Carboplatin	0.00396	3
*SNRK*	Cisplatin	0.00479	3
*SNRK*	Daunorubicin	0.02429	3
*SULF2*	Carboplatin	0.00002	20
*SULF2*	Cisplatin	0.00053	20
*SULF2*	Daunorubicin	0.00014	20
*SULF2*	Etoposide	0.00000	20
*TBC1D22A*	Cisplatin	0.03807	22
*TBL1XR1*	Carboplatin	0.04028	3
*TFDP1*	Cisplatin	0.04967	13
*TNK2*	Carboplatin	0.01901	3
*TNK2*	Daunorubicin	0.00431	3
*TNK2*	Etoposide	0.01159	3
*TYRO3*	Etoposide	0.02840	15
*UPK3B*	Cisplatin	0.02839	7
*WDR90*	Daunorubicin	0.02575	16
*WDR90*	Etoposide	0.00111	16
*ZDHHC4*	Daunorubicin	0.02463	7
*ZFAND2A*	Daunorubicin	0.00227	7
*ZFAND2A*	Etoposide	0.03567	7
*ZNF277*	Etoposide	0.04521	7
*ZNF331*	Carboplatin	0.02122	19
*ZNF664*	Carboplatin	0.04722	12

Transcripts are *cis*-regulated by CNVs identified from whole-genome sequencing.

### Drug susceptibility-associated CNVs are independent of drug susceptibility-associated SNPs

We investigated to what extent the CNVs associated with cellular sensitivity to chemotherapeutic agents may already be interrogated by SNP-based GWAS through linkage disequilibrium [6]. We found that the top CNV (*CNVR1616.1*) associated with carboplatin IC_50 _(*P *= 5 × 10^-4^) is not well-tagged by SNPs. Indeed, the best proxy SNP for this CNV on chromosome 3 is *rs967422 *(at *r*^*2 *^= 0.075). We found that the same CNV is also associated with cisplatin IC_50 _(*P *= 6.5 × 10^-3^). Another cisplatin-associated CNV (*P *= 5.5 × 10^-3^), *CNVR7870.1*, is also not well-tagged; the best proxy SNP, *rs915049*, tags the CNV at a low *r*^*2 *^= 0.11. In each case, the best proxy SNP, in contrast to the 'tagged' drug susceptibility-associated CNV, shows no evidence of being associated with cellular sensitivity to the drug even at the nominal threshold of *P *= 0.05.

In the case of the topoisomerase II inhibitors, of the CNVs showing association with both etoposide and daunorubicin (*P *< 0.05), we found two - *CNVR7205.1 *and *CNVR3293.1 *- that are only modestly tagged (by *rs563079 *at *r*^*2 *^= 0.77 and *rs17166803 *also at *r*^*2 *^= 0.77, respectively). Neither *rs563079 *nor *rs17166803 *is associated with etoposide or daunorubicin IC_50_. In contrast, *CNVR2930.1*, which is one of two etoposide-associated CNVs predicting the expression of *CCND1 *(expression *P *= 2.4 × 10^-7^), is perfectly tagged (*r*^*2 *^= 1) by *rs9500270*. We identified a daunorubicin-associated CNV (*CNVR2766.1*; *P *= 3.7 × 10^-3^) for which the best proxy SNP, *rs10484327*, tags the CNV at only *r*^*2 *^= 0.11.

### PACdb: a database for cell-based pharmacogenomics

**Figure 2:**
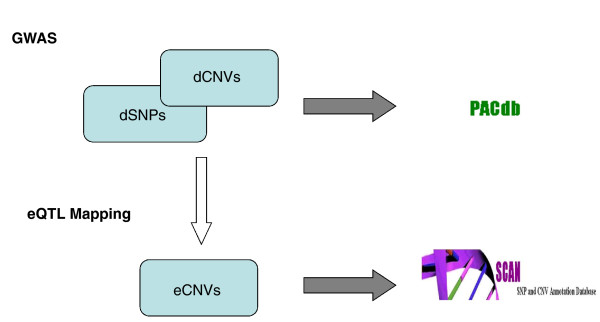
**A schematic of the approach to the discovery of drug susceptibility-associated CNVs**. We added to PACdb the CNV associations with cellular sensitivity to the chemotherapeutic agents. Note that some drug-associated CNVs (dCNVs) are poorly interrogated by SNPs and are thus independent of drug-associated SNPs (dSNPs). SCAN includes the results of eQTL mapping of CNVs to transcriptional expression.

PACdb [24] is a large-scale, publicly available genomic database, which to date holds the results of our SNP-based GWAS on the following chemotherapeutic agents: carboplatin, cisplatin, etoposide, daunorubicin, and cytarabine. PACdb implements a structured repository for incorporating other datasets, including information on other drugs, gene expression profiling, and cellular phenotypes. GWAS were initially conducted using SNP genotype data made available by the International HapMap project. We expanded PACdb to include the results of our CNV-based GWAS on carboplatin, cisplatin, etoposide, and daunorubicin. Furthermore, the results of eQTL mapping of HapMap CNVs to transcriptional expression are made available in the eQTL repository SCAN. Figure 2 shows a schematic diagram of our approach to the discovery of CNVs associated with sensitivity to drug and to the identification of such CNVs that act as eQTLs; it also illustrates the genomic resources we have made publicly available to the scientific community.

### CNVs and drug classes

**Figure 3:**
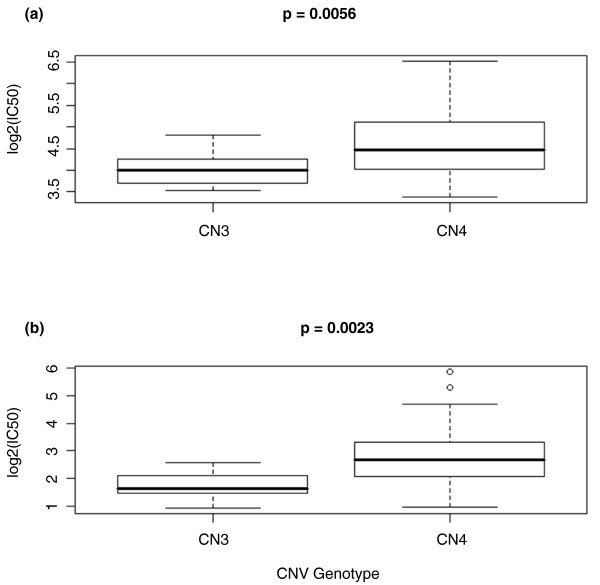
**A CNV associated with cellular sensitivity to both carboplatin and cisplatin**. We identified an amplification CNV, *CNVR7826_full *on chromosome 20, that is associated with both carboplatin sensitivity (a) (*P *= 0.0056) and cisplatin sensitivity (b) (*P *= 0.0023). The x-axis is the copy number (3 or 4) for the CNV; the y-axis is the log_2_-transformed IC_50_.

We evaluated to what extent the top CNV associations for a given drug would overlap with the top CNV associations for another drug belonging to the same chemotherapeutic drug class, defined in terms of mechanism of action. At the suggestive threshold of *P *< 0.05, of the CNVs showing association with carboplatin IC_50_, 16% (*n *= 11) were also associated with cisplatin IC_50. _Thus, we see a significant overlap (*P *= 7.7 × 10^-10^) between the (two) sets of CNVs associated with cellular sensitivity to the platinating agents. Figure 3 illustrates a duplication (*CNVR7826_full *on chromosome 20) that is associated with sensitivity to carboplatin (Figure 3a) and to cisplatin (Figure 3b); note that the observed genotype associations with the platinums have concordant direction. Furthermore, the CNV is an eQTL predicting the expression of *GSR *(*P *= 4.67 × 10^-5^) and *SPARC *(*P *= 4.70 × 10^-5^). Remarkably, the expression levels of these target mRNAs, *GSR *(*P *= 0.045) and *SPARC *(*P *= 0.004), are correlated with carboplatin IC_50_; similarly, *GSR *(*P *= 0.005) and *SPARC *(*P *= 0.005) are correlated with cisplatin IC_50_. Glutathione reductase (*GSR*) has been implicated in several studies of platinum sensitivity [25,26].

In the case of the topoisomerase II inhibitors, 12% of the etoposide-associated CNVs were found to associate with daunorubicin IC_50_, and the observed overlap is still quite significant (*P *= 2.7 × 10^-10^). The slightly greater percentage of overlap for the platinating agents is not due to higher phenotypic correlation (platinating agents (r = 0.52) versus topoisomerase II inhibitors (r = 0.69)).

### Real-time PCR validation

**Figure 4:**
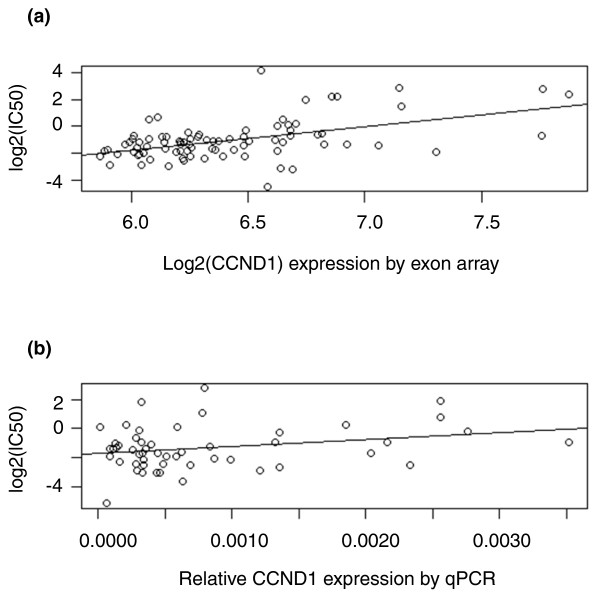
**The relationship between *CCND1 *expression and etoposide IC**_**50 **_**in the discovery set and in an independent replication set of 52 CEPH LCLs**. **(a) **Discovery set; (b) independent replication set of 52 CEPH LCLs. Panel (b) describes the expression- IC_50 _relationship using real-time expression; the relationship is consistent with that in the discovery set of CEU samples.

We sought additional experimental support for the genes targeted by multiple CNVs associated with drug susceptibility. We identified two etoposide-associated CNV eQTLs that share *CCND1 *as a target gene (expression *P *= 2.4 × 10^-7^). The over-expression of *CCND1 *has been shown to be associated with the up-regulation of the GST-π gene, increasing the sensitivity of a cancer cell line to etoposide [27]. We found *CCND1 *expression to be significantly correlated with etoposide IC_50 _(*P *= 7.8 × 10^-6^) in the CEU samples. After multiple testing correction, the gene remained significant (q-value = 0.0027). We subsequently conducted functional validation of the role of *CCND1 *expression in altering sensitivity to etoposide by performing real-time quantitative-PCR assays in an independent set of 52 CEPH LCLs (Figure 4; see Table S1 in Additional file 1 for the real-time PCR data on *CCND1*). Consistent with the direction of effect in the CEU samples, increased *CCND1 *mRNA levels resulted in increased IC_50 _(*P *= 0.05) in the validation set. Thus, increasing *CCND1 *expression confers resistance to etoposide.

## Discussion

Understanding in a comprehensive manner the genetic risk factors contributing to variation in drug response is a crucial component of the realization of personalized medicine. The drugs evaluated in our study are widely used in the treatment of many cancer types, including ovarian, colorectal, testicular, and lung; all are associated with particular toxicities and resistance. Although SNPs have long been used in association studies to elucidate the effect of genetic polymorphisms on drug response, CNVs have been relatively understudied. Recent genome-wide surveys of CNVs have now established that these structural variants are a common phenomenon in the human genome [5]. With rapid advances in methods that facilitate their assay and analysis, variation in copy number for genes encoding drug metabolizing enzymes has been increasingly implicated for their dramatic consequences on responsiveness to drugs. Such CNVs have been observed to alter gene dosage and are thus likely to play an important role in determining drug efficacy or toxicity.

In this study, we set out to utilize recent developments in the assay of CNVs in recent population-scale projects, including an extensive comparative genomic hybridization-based catalog of CNVs [4] and a map of structural variants based on whole genome DNA sequencing data (the 1000 Genomes Project) [14], in order to evaluate the role of CNVs in cellular sensitivity to chemotherapeutic agents. The cell lines for the samples express a sizable part of the genome [28], thus enabling the investigation of genes represented in biologically relevant pathways. While the cancer genome is clearly necessary for understanding chemotherapeutic response, the importance of germline genetic variation in drug sensitivity has also been consistently demonstrated [15].

For each drug included in our study, we found that the top associated CNVs are more likely to act as eQTLs and predict transcript levels than minor allele frequency (MAF)-matched SNPs. The overlap of the drug susceptibility-associated CNVs with expression-associated CNVs (eCNVs) is greater than is expected, based on simulation studies. Consistent with a previous report [6], CNVs associated with cellular sensitivity to drug treatment are not likely to overlap exons, suggesting that they act not to disrupt coding sequence but to regulate gene expression. The high proportion of eQTLs among the CNVs associated with cellular sensitivity to each of the drugs further supports the hypothesis that these CNVs mediate their phenotypic consequences through their effect on the transcriptome. Genome-wide studies of pharmacologic phenotypes, such as response to antineoplastic agents, may benefit from studies of CNVs as eQTLs.

This study, to our knowledge, is the first comprehensive genome-wide study of the effect of CNVs, from the most extensive array-based and sequencing-based surveys of these structural variants, on pharmacologic phenotypes. In contrast to a recent disease susceptibility study that concluded that most CNVs that are well-typed have been indirectly explored by SNP studies [6], we found a number of CNVs associated with drug sensitivity that are independent of SNPs. These CNVs therefore constitute novel genetic variations that have not been previously interrogated by SNP-based GWAS of pharmacologic phenotypes. Our discovery of drug susceptibility-associated variations, in the form of CNVs, that are independent of previous SNP findings and that show evidence for altering gene expression as eQTLs, suggests that CNVs should be included in comprehensive pharmacogenomic studies.

Candidate pharmacogenetic studies on drug metabolism-related genes, namely *CYP2D6*, *CYP2A6*, *SULT1A1 *and *GSTM1*, have documented the effect of CNVs on gene activity. Our results strongly support the necessity of integrating both SNP and CNV data to tighten the genotype-phenotype gap in pharmacogenetic studies. While the functional validation we conducted in this study may not allow robust predictions, the functional characterization of the effect of *CCND1 *mRNA level on cellular sensitivity to etoposide underscores the importance of considering the role of the transcripts that are the targets of drug susceptibility-associated CNVs (acting as eQTLs) in conferring drug susceptibility.

We found a significant overlap (*P *= 7.7 × 10^-10^) between the CNVs associated with cisplatin and carboplatin. Platinating agents share a similar mechanism of therapeutic action and interact with DNA to form interstrand and intrastrand cross-links, leading to cytotoxic DNA lesions and eventually apoptosis-induced cell death. Our findings strongly support the hypothesis that CNV-based mechanisms play a crucial role in determining platinum sensitivity. Particularly, we identified a duplication that is associated with cellular sensitivity to both carboplatin and cisplatin. Furthermore, the CNV predicts the expression of glutathione reductase (*GSR*), a gene that has been the subject of several studies on cisplatin sensitivity [26,30]. The glutathione pathway is involved in the metabolism of platinum compounds, which are subject to inactivation by glutathione conjugation [27].

A significant level of overlap is also observed with the topoisomerase II inhibitors. Daunorubicin is a DNA intercalator that indirectly interacts with topo II while etoposide binds directly to the enzyme. We identified 14 CNVs associated with both etoposide and daunorubicin at *P *< 0.05. The extent of overlap between the platinating agents (as well as between the topoisomerase II inhibitors) is significantly higher than the level of overlap across drug classes (7%).

There is a general caveat to our findings concerning the set of CNVs included in this analysis. The CNVs tested for association with cellular sensitivity to drugs may be biased towards genotypeable variants; consequently, many highly complex regions may have been excluded. Furthermore, our study makes no assertions about low frequency variants. Nevertheless, our findings represent the most comprehensive study of the effect of common CNVs, from the most extensive map of these variants available, on chemotherapeutic susceptibility to a wide array of drugs.

Finally, we provide the results of our genome-wide study of CNVs and sensitivity to chemotherapeutic agents in a publicly available online database, PACdb. Analysis results on our cell-based model are easy to query, which should allow investigators to utilize the resource as a discovery platform or as a validation tool for clinical observations.

## Conclusions

Our study identified CNVs that predict cellular sensitivity to an array of chemotherapeutic agents of heterogeneous molecular therapeutic action. Importantly, several of the most significant CNV-drug associations are independent of SNPs; thus, these CNVs provide genetic variations that have not been previously explored by SNP-based GWAS of pharmacologic phenotypes. Furthermore, our findings show that pharmacogenomic studies may be greatly enhanced by studies of CNVs as eQTLs. Target genes of CNVs, especially those associated with multiple independent CNVs associated with drug response, provide robust gene expression signatures of chemotherapeutic susceptibility.

## Materials and methods

### *In vitro *cellular sensitivity to chemotherapeutic agents

We obtained unrelated HapMap phase II CEU (Utah residents with ancestry from northern and western Europe) samples from Coriell Institute for Medical Research (Camden, NJ, USA). Cell lines were maintained in RPMI 1640 media supplemented with 15% fetal bovine serum (Hyclone, Logan, UT, USA) and 1% l-glutamine. The cell lines were passaged three times per week at a concentration of 350,000 cells/ml at 37°C in a 95% humidified 5% CO_2 _atmosphere. Cellular sensitivity to drugs was measured in these cell lines with increasing concentrations of drug (from carboplatin, cisplatin, daunorubicin, and etoposide). Cell growth inhibition was evaluated using the alamarBlue™ assay (BioSource International Inc., Camarillo, CA, USA), as previously described [21]. IC_50 _(the concentration required to inhibit 50% of cell growth) was determined by curve fitting of percent cell survival against concentrations of the drug.

### A catalog of CNVs

Recent population-based surveys have mapped thousands of CNVs with increasing resolution. Such surveys have opened up approaches for modeling the relationship between structural variation and complex traits. Efforts to catalog these CNVs are necessary to clarify the functional impact of these variants. Here we utilize the recent comprehensive survey of CNVs [4] larger than 1 kb in size in the human genome, assayed in human LCLs from CEU (Utah residents with ancestry from northern and western Europe) samples. To investigate further the effect of deletions and to confirm our findings on the role of drug-associated CNVs as eQTLs, we studied the relationship between *cis*-regulated transcripts (from Gencode/ENCODE functional annotation) and cellular sensitivity to chemotherapeutics from a recent comprehensive study based on whole genome sequencing data of the 1000 Genomes Project [14], which mapped CNVs of 50 bp or larger in size at nucleotide resolution.

### Association analysis of CNVs or transcript levels with cellular sensitivity to drugs

For each CNV, genotypes were tested for association with cellular sensitivity to each of the drugs separately. Linear regression was performed between the copy number genotype (as the independent variable) and log_2_-transformed IC_50 _(as the dependent variable). Analysis was done in the statistical computing and graphics software R; the *lm *function was used to fit linear models.

Similarly, to examine the relationship between transcript level and drug-induced cellular sensitivity, a linear model was constructed for each drug, as previously described [19], between log_2_-transformed gene expression and log_2_-transformed IC_50_. Generally, for multiple testing adjustment, FDRs were calculated using the q-value [23] package in R. Unless otherwise stated, an FDR cutoff <0.05 was used as the statistical significance threshold.

### Mapping CNVs as expression quantitative trait loci

SCAN [29] is an online database that makes publicly available the results of our eQTL studies, initially on single base polymorphisms. Global mRNA expression was assayed using the Affymetrix GeneChip Human Exon 1.0 ST Array [30]. To map CNVs as genomic loci influencing the transcriptome, we conducted linear regression on over 13,000 transcript clusters and the set of CNVs identified in the HapMap LCLs [31].

### Simulation studies

We performed simulations to evaluate enrichment for eQTLs among the CNVs associated with cellular sensitivity to the drugs included in our study. To empirically generate the null distribution, we randomly generated sets of SNPs of matching minor allele frequency as the original list of CNVs (see Figure S1 in Additional file 2 for MAF distribution of the biallelic CNVs included in our study), as previously described [32]. To enable us to perform simulations conditional on MAF, we constructed non-overlapping MAF bins, each of width 0.05, using the MAFs of the SNPs in the HapMap CEU samples. The null sets were drawn from the combined platform SNPs (Affymetrix 6.0 and Illumina 1M) as well as from the entire set of HapMap CEU SNPs. The observed count is then compared to the empirically generated distribution to get an empirical *P*-value for the enrichment.

### Functional validation

We obtained 52 unrelated non-HapMap CEPH (Centre d'Etude du Polymorphisme Humain) samples (independent of the discovery cohort consisting of HapMap CEU samples) from Coriell Institute for Medical Research. Cellular sensitivity to etoposide phenotype was quantified as described above with increasing concentrations of etoposide treatment (0.02 μM, 0.1 μM, 0.5 μM, and 2.5 μM for 72 hours). IC_50 _was determined for each cell line. *CCND1 *mRNA levels were evaluated using a real-time quantitative PCR assay in the samples using TaqMan Gene Expression Assays (Applied Biosystems, Foster City, CA, USA) on the Applied Biosystems 7500 real-time PCR system. Primer/probes were obtained from Applied Biosystems. The human beta 2M (*huB*_*2*_*M*, beta-2 microglobulin; NM_004048; Applied Biosystems catalog number 4326319E) was used as endogenous control. Relative quantification of gene expression utilized the 2 ^(-ΔΔCt) ^method [33].

## Abbreviations

bp: base pair; CNV: copy number variant; eQTLs: expression quantitative trait loci; FDR: false discovery rate; GST: Glutathione S-transferase; GWAS: genome-wide association studies; LCL: lymphoblastoid cell line; MAF: minor allele frequency; PCR: polymerase chain reaction; SNP: single nucleotide polymorphism.

## Competing interests

The authors declare that they have no competing interests.

## Authors' contributions

ERG conceived the study. ERG, RSH, MED, and NJC designed the experiments and the analyses and wrote the manuscript. ERG analyzed the data. All authors read and approved the final manuscript.

## Supplementary Material

Additional file 1 **Real-time PCR data on *CCND1***. The table lists the real-time PCR. values for *CCND1 *expression, as measured in the independent set of 52 cell lines. Click here for file

Additional file 2 **The minor allele frequency distribution of the biallelic CNVs included in our study**. The plot is a histogram of the minor allele frequency of the biallelic CNVs that were evaluated in our study. Click here for file
